# Fully automated volumetric modulated arc therapy planning for locally advanced rectal cancer: feasibility and efficiency

**DOI:** 10.1186/s13014-023-02334-0

**Published:** 2023-09-05

**Authors:** Kouta Hirotaki, Kento Tomizawa, Shunsuke Moriya, Hajime Oyoshi, Vijay Raturi, Masashi Ito, Takeji Sakae

**Affiliations:** 1 Doctoral Program in Medical Sciences, Graduate School of Comprehensive Human Sciences, University of Tsukuba, Ibaraki, Japan; 2 Department of Radiological Technology, National Cancer Center Hospital East, Chiba, Japan; 3 Department of Radiation Oncology, National Cancer Center Hospital East, 6-5-1, Kashiwanoha, 277-8577 Kashiwa, Chiba Japan; 4 Faculty of Medicine, University of Tsukuba, Ibaraki, Japan; 5 Department of Radiation Oncology, Apollomedics Hospital, Lucknow, India

**Keywords:** Rectal cancer, Volumetric modulated arc therapy, Automated planning, Dosimetry, Raystation, Work efficiency

## Abstract

**Background:**

Volumetric modulated arc therapy (VMAT) for locally advanced rectal cancer (LARC) has emerged as a promising technique, but the planning process can be time-consuming and dependent on planner expertise. We aimed to develop a fully automated VMAT planning program for LARC and evaluate its feasibility and efficiency.

**Methods:**

A total of 26 LARC patients who received VMAT treatment and the computed tomography (CT) scans were included in this study. Clinical target volumes and organs at risk were contoured by radiation oncologists. The automatic planning program, developed within the Raystation treatment planning system, used scripting capabilities and a Python environment to automate the entire planning process. The automated VMAT plan (auto-VMAT) was created by our automated planning program with the 26 CT scans used in the manual VMAT plan (manual-VMAT) and their regions of interests. Dosimetric parameters and time efficiency were compared between the auto-VMAT and the manual-VMAT created by experienced planners. All results were analyzed using the Wilcoxon signed-rank sum test.

**Results:**

The auto-VMAT achieved comparable coverage of the target volume while demonstrating improved dose conformity and uniformity compared with the manual-VMAT. V30 and V40 in the small bowel were significantly lower in the auto-VMAT compared with those in the manual-VMAT (p < 0.001 and < 0.001, respectively); the mean dose of the bladder was also significantly reduced in the auto-VMAT (p < 0.001). Furthermore, auto-VMAT plans were consistently generated with less variability in quality. In terms of efficiency, the auto-VMAT markedly reduced the time required for planning and expedited plan approval, with 93% of cases approved within one day.

**Conclusion:**

We developed a fully automatic feasible VMAT plan creation program for LARC. The auto-VMAT maintained target coverage while providing organs at risk dose reduction. The developed program dramatically reduced the time to approval.

## Background

Colorectal cancer causes more than 1.9 million deaths worldwide annually and its mortality rate ranks second among all cancers, and rectal cancer accounts for approximately one-third of all colorectal cancers [1]. The treatment outcome of patients with locally advanced rectal cancer (LARC) has been unsatisfactory because of the lack of locoregional control achieved with only surgical resection [2]. The introduction of total mesorectal excision and neoadjuvant chemoradiation for LARC has improved locoregional control [3, 4]. Three-dimensional conformal radiotherapy is the most common radiotherapy technique [5]. However, it is difficult to spare organs at risk (OAR) such as the small bowel and the bladder while keeping the dose to the target in three-dimensional conformal radiotherapy for LARC [6]. Recently, volumetric modulated arc therapy (VMAT) has been widespread and plays an essential role in radiation therapy for LARC. While VMAT provides better dose distribution compared with three-dimensional conformal radiotherapy, VMAT planning and validation require additional effort [7–9]. Furthermore, the quality of the VMAT plan depends on the experience and skill of the individual planner [10–13].

Several studies have reported on automatic planning aimed at reducing planner labor and standardizing plan quality [14]. Currently, the mainstream methods of automatic planning are knowledge-based planning, protocol-based automatic iterative optimization, and multicriteria optimization [15]. Knowledge-based planning predicts dose-volume histograms by learning a large number of clinically accepted plans. While this approach allows for efficient and standardized treatment planning, the quality of the dose distribution depends on the input data [16, 17]. The protocol-based automated iterative optimization automatically updates constraints toward clinical goals but requires experience in setting up calculations [18–20]. Multicriteria optimization is a method of simultaneously optimizing multiple scenarios with different trade-off relationships. This method can provide a desired plan from multiple completed plans, but the calculation time increases as the number of scenarios increases [21, 22]. In contrast, script-based auto planning does not require a pre-learning step or initial input. It has the potential to achieve higher accuracy and speed than commercial auto-planning systems. However, previous reports of script-based auto plans using Raystation are still scarce [23, 24].

In this study, we developed a fully automatic VMAT planning program for LARC using the development environment of Raystation with a high degree of freedom. We evaluated the feasibility of our automated planning through dose indices and time efficiency in comparison with manual planning.

## Methods

### Patient and image dataset

The VMAT plans for 26 patients with LARC treated at our institution from April 2020 to March 2022 and the computed tomography (CT) scans were included in this study (Table 1). The automated VMAT plan (auto-VMAT) was created by our planning program using the 26 CT scans used in the manual VMAT plan (manual-VMAT) and their regions of interest (ROIs). Simulation CT images were acquired using Aquilion One (Canon Medical Systems, Tochigi, Japan), and the image slice thickness was 2 mm. A 400 ml water load and a waiting time of 30–50 min were used for patient pretreatment to fill the bladder before the CT scan. The contents of the study, including the investigation procedure and the handling of patient information, were approved by the institutional review board of the National Cancer Center Hospital East (IRB No. 2018-076).

**Table 1:** Patient characteristics

Patient	Age	Sex	PTV-initial volume[cm^3^]	PTV-boost volume[cm^3^]	Tumor site	TNM*
1	45	Male	674.20	64.82	Ra	T3N2M0
2	47	Male	835.88	314.1	RS	T4N2M0
3	45	Male	737.86	198.49	Rb	T4N0M0
4	69	Male	960.23	498.86	Ra-Rb	T3N3M0
5	66	Male	1157.58	149.31	Rb	T3N3M0
6	43	Male	1082.17	309.49	Rb	T4N3M0
7	32	Male	1368.73	400.46	RS	T4N2M0
8	60	Male	909.71	475.31	Ra-Rb	T4N1M0
9	45	Male	834.62	241.53	Rb	T3N3M0
10	66	Male	1055.13	224.14	Rb	T3N3M0
11	47	Male	1252.46	371.59	RS-Ra	T3N3M0
12	80	Male	884.12	249.85	Rb	T3N3M1
13	40	Male	1011.44	265.38	Rb	T3N1M0
14	63	Male	636.49	171.02	Rb	T3N0M0
15	58	Male	1133.94	242.09	Rb	T2N0M0
16	53	Male	1110.99	285.41	RS	T3N0M0
17	70	Male	1120.22	143.99	RS-Ra	T4N0M0
18	59	Male	976.78	295.41	Rb	T3N1M0
19	71	Male	759.86	225.12	Rb	T3N0M0
20	50	Female	676.39	219.22	Rb	T3N0M0
21	63	Female	724.30	242.85	Rb	T3N0M0
22	58	Female	1039.00	289.04	Rb	T4N1M1
23	52	Male	895.36	255.85	Ra-Rb	T3N2M0
24	77	Male	888.98	285.63	Ra-Rb	T3N0M0
25	55	Male	824.67	249.67	Rb	T3N1M0
26	67	Female	980.13	237.07	Ra-Rb	T4N1M1

Abbreviations: PTV, planning target volume; Ra, which is the segment of the rectum from the height of the inferior border of the second sacral vertebra to the peritoneal reflection; Rb, which is the segment of the rectum located below the peritoneal reflection; or P, which is the anal canal; Rs, which is the segment from the height of the sacral promontory to the inferior border of the second sacral vertebra [27]. * 8th edition of UICC [28]

### Contouring

The clinical target volume (CTV), the planning target volume (PTV), and OARs including bladder, small intestine, and femoral heads were delineated by radiation oncologists. The CTV of the initial plans included the primary tumor, metastatic lymph nodes, and relevant regional lymph nodes (mesorectum, internal iliac, obturator, presacral, and external iliac depending on T-stage). The CTV of the boost plans was created based on the primary tumor. A 0.5 cm margin in anterior-posterior and right-left directions and 2 cm margin in craniocaudal directions were given to make the CTV of both initial and boost plans. No margin was given in all directions for metastatic lymph nodes to create CTV. An expansion with a margin of 0.5 cm was given in all directions for CTV to make the PTV.

### Treatment Planning and clinical goals

The prescribed doses were 45 Gy and 5.4 Gy for initial and boost plans, respectively. All treatment plans were generated with 10-MV photon beams; the collimator angle was arbitrary for the manual-VMAT and 355° for the auto-VMAT. A single full 360° coplanar arc was adopted for both initial and boost plans. The isocenter was set at the center of PTV for the initial plan (PTV initial) and PTV for the boost plan (PTV boost). Treatment planning was performed using Raystation (RaySearch Laboratories AB, Stockholm, Sweden). The manual-VMAT was created by four experienced planners. Both the auto-VMAT and manual-VMAT were calculated using the Collapsed Cone V5.3 algorithm. Table 2 shows the clinical goals for dose indices. The goals of the PTV coverage were to reach D93 = 98% in both the initial and boost plans. Dose constraints for PTVs and OARs were set with reference to RTOG 0822 [25]. Exceeding the maximum dose clearance to the small bowel and bladder was clinically allowed considering the overlap with the PTV. Adherence to OAR dose was prioritized in determining clinical acceptability.

**Table 2:** Clinical Goal

			Criteria
PTV			
	D93		> 98%
	D2		< 110%
Small bowel			
	V35		< 230 cc
	V40		< 130 cc
	V45		< 90 cc
Bladder			
	V40		< 55%
	V45		< 30%
	V50		<= 0%
Femoral heads			
	V40		< 65%
	V45		< 45%
	V50		<= 0%

Abbreviations: PTV, planning target volume; VX, the percentage of the organ volume that received X Gy or more; DX, dose received by the X% of the volume

### Script-based Auto Planning

**Fig. 1:**
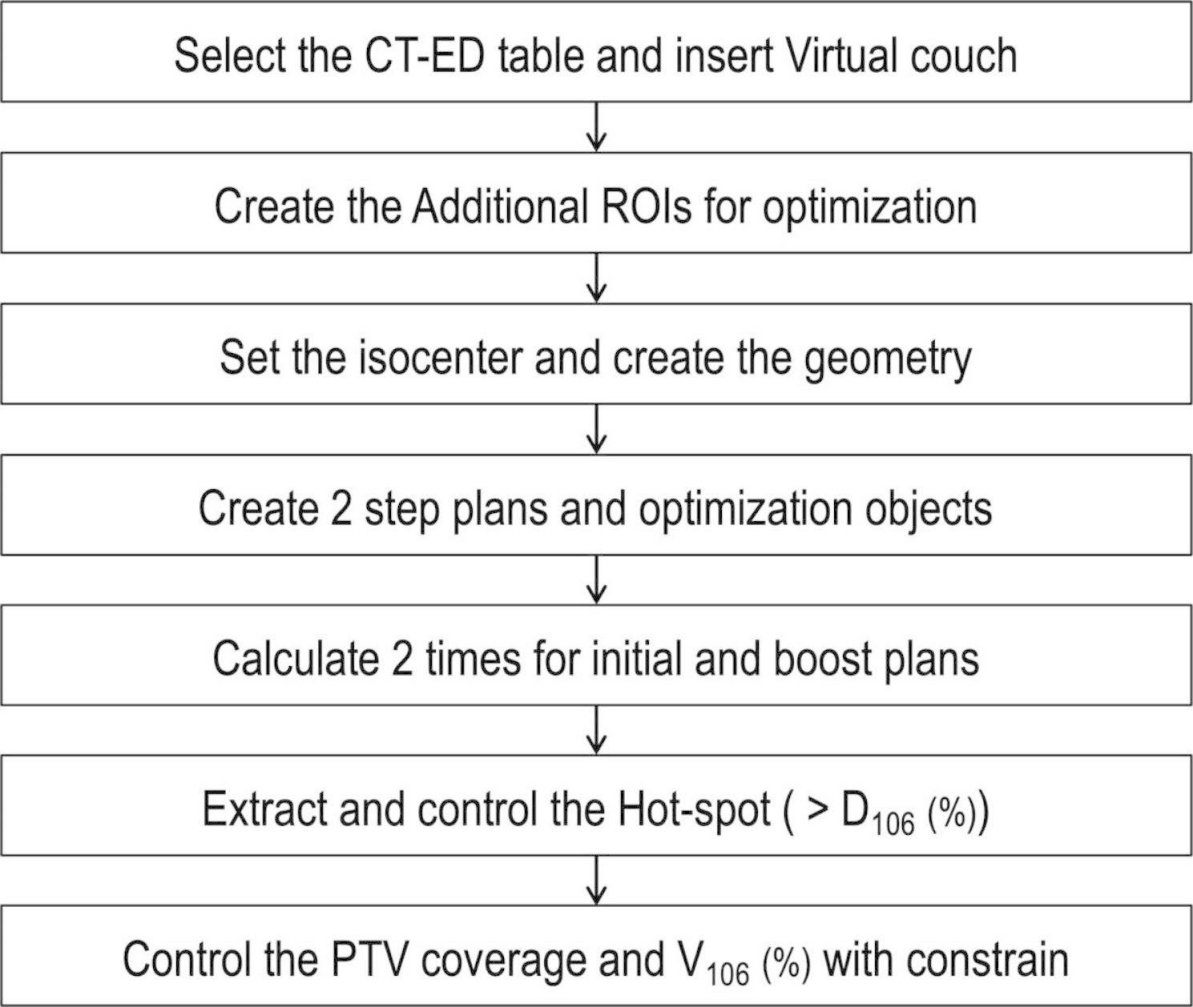
The workflow of the script-based fully automatic planning program

Figure 1 shows the overview of the automatic planning system. The automatic planning system was built with scripting capabilities within Raystation and a Python (version 3.6) environment. The consistency of the defined ROI name and physician contouring was checked before starting the automatic program. After starting the program, the CT electron density conversion table was automatically selected, and a virtual couch was inserted. ROIs for optimization are automatically generated. The program automatically created the ROIs necessary to generate the plan by referencing the names of the ROIs imported from the contour data. Additional ROIs for OARs were created by removing the overlap with the PTV from the bladder and small bowel. Additional ROIs to control the dose distribution shape were created by expanding from the PTV in the abdominal direction. The additional ROIs generated by this method can easily form a bowel bag-like shape without depending on the PTV shape and size to reduce the OAR dose. Prescription dose and geometry were automatically input. The isocenter was set at the center of PTV initial and PTV boost. We adopted a dose fall off constraint to each OAR to achieve short-term optimization. Objects for dose constraint were automatically input, and optimization was performed two times for initial and boost plans. The first dose distribution was created; next, hot-spots were extracted from the distribution, and optimization was performed three times with additional dose objects for hot-spot correction. Constrained objects were added to achieve the clinical goal for PTV and optimization was performed. The ROI display and dose distribution color bar were then automatically set, and physicians checked the dose distribution.

### Evaluation

Dose indices for PTVs and OARs were compared in the manual-VMAT and auto-VMAT. OAR doses were evaluated for the sum of the initial and boost plans. In dose indices for OARs, the mean dose and maximum dose were evaluated for the bladder; V30, V40, and maximum dose for small bowel; and mean dose and maximum dose for femoral heads. D93, D2, conformity index (CI), and homogeneity index (HI) were evaluated for initial and boost PTVs. CI and HI are defined in formulas 1 and 2.

1 $${\rm{CI}}\,{\rm{ = }}\,{{\rm{(TV\_PIV)}}^{\rm{2}}}{\rm{/}}\,{\rm{(TV}}\,{\rm{ \times }}\,{\rm{PIV)}}$$

Where TV_PIV is the volume of the target covered by the prescription isodose volume (PIV), TV is the target volume, and PIV is the volume covered by the prescribed isodose.

2 $$HI{\rm{ }} = {\rm{ }}\left( {D2{\rm{ }} - {\rm{ }}D98} \right){\rm{ }}/{\rm{ }}D\_prescription$$

Where D2 is the dose received by 2% of the target volume, D98 is the dose received by 98% of the target volume, and D_ prescription is the prescribed dose.

To evaluate work efficiency, we measured the time required for planning by our program and the number of days required until plan approval. Days to plan approval was defined as the number of days from the time the physician finished contouring to the time the plan was approved by physicians. The time and days at which contouring, plans, and approvals were completed were measured using the in-house developed system.

### Plan specific quality assurance

Patient-specific quality assurance (QA) was conducted for all auto-VMATs and manual-VMATs using Delta4® (ScandiDos, Uppsala, Sweden). Criteria of the gamma passing rate were a dose difference of 3% and a distance to agreement of 2 mm (3%/2mm). The gamma pass rate of greater than 98% was the threshold to indicate robust delivery in this study. Modulation Complexity Score (MCS) was calculated to assess the clinical feasibility of the auto-VMAT using Simple MU Analysis® (Triangle Products Co.Ltd, Chiba, Japan). MCS range 0.0–1.0 and the closer to 0 means the more complex the plan [26].

### Statistics

All results were analyzed using the Wilcoxon signed-rank sum test. p < 0.05 indicated statistical significance. The analyses were two-sided and performed using the exactRankTests packages in R v.4.2.2.

## Results

### Dosimetry Comparison

**Fig. 2:**
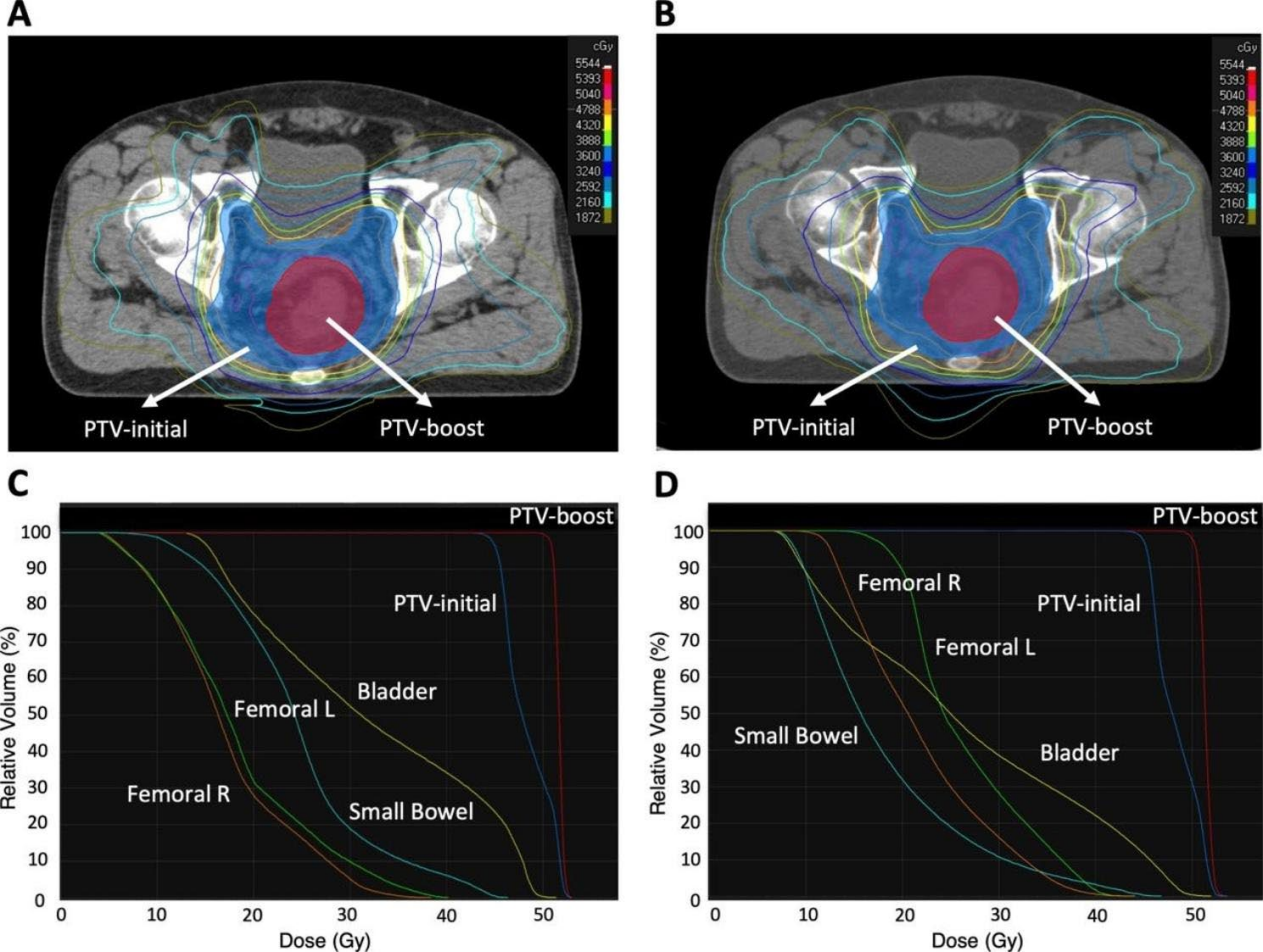
Comparison of representative dose distributions and dose-volume histograms (DVH) between two types of Volumetric modulated arc therapy (VMAT) plans. Dose distributions on an axial slice and DVHs in the plan using the manual VMAT (A, C) and automated VMAT (B, D)

A comparison of the representative dose distributions and dose-volume histograms between auto-VMAT and manual-VMAT is shown in Fig. 2A–D. Table 3 shows the comparison of the manual-VMAT and auto-VMAT for PTV coverage and dose to OARs. The D93 of the auto-VMAT was significantly higher in both the initial and boost plans (p = 0.019 and < 0.001, respectively). The CI value of the manual-VMAT was significantly higher in the initial plan (p = 0.002). The HI value of the auto-VMAT was significantly higher for both initial and boost plans (p = 0.003 and < 0.001, respectively). The V30 and V40 of the small bowel of the auto-VMAT were significantly lower compared with the manual-VMAT (p < 0.001 and < 0.001, respectively). No significant difference in the maximum dose of the small bowel was observed between the manual-VMAT and auto-VMAT. The mean dose of the bladder of the auto-VMAT was significantly lower than the manual-VMAT (p = < 0.001). No significant difference was observed for the maximum dose of the bladder between the manual-VMAT and auto-VMAT (p = 0.829). The mean and maximum dose of femoral heads of the manual-VMAT was significantly lower for both the right (p < 0.001 and < 0.001, respectively) and left (p < 0.001 and = 0.043, respectively) sides.

**Table 3:** Comparison of dose indices

		Manual-VMAT (Mean ± SD)	Auto-VMAT (Mean ± SD)	*P* values
**PTV initial**				
	D98 (%)	95.2 ± 1.6	95.3 ± 1.6	0.748
	D93 (%)	99.6 ± 0.2	99.8 ± 0.2	0.019
	D2 (%)	104.6 ± 0.5	104.6 ± 0.4	0.943
	CI	0.83 ± 0.06	0.78 ± 0.03	0.002
	HI	0.72 ± 0.05	0.76 ± 0.04	0.003
**PTV boost**				
	D98 (%)	95.7 ± 4.5	98.7 ± 0.9	< 0.001
	D93 (%)	97.8 ± 3.5	99.9 ± 0.2	< 0.001
	D2 (%)	104.3 ± 0.8	103.5 ± 0.4	< 0.001
	CI	0.79 ± 0.08	0.82 ± 0.05	0.222
	HI	0.69 ± 0.17	0.80 ± 0.07	< 0.001
**OAR for plan sum**				
Small bowel				
	Dmax (Gy)	48.6 ± 3.8	49.3 ± 3.6	0.053
	V30 (%)	29.8 ± 16.7	21.5 ± 12.1	< 0.001
	V40 (%)	16.9 ± 12.8	12.6 ± 8.6	< 0.001
Bladder				
	Mean dose (Gy)	30.4 ± 3.6	24.4 ± 4.4	< 0.001
	Dmax (Gy)	50.3 ± 2.0	50.2 ± 2.4	0.829
	V50 (%)	1.6 ± 3.0	2.0 ± 3.0	0.776
Right femoral head				
	Mean dose (Gy)	18.1 ± 2.5	21.7 ± 3.4	< 0.001
	Dmax (Gy)	39.0 ± 3.5	41.5 ± 3.0	< 0.001
Left femoral head				
	Mean dose (Gy)	17.6 ± 3.5	21.8 ± 3.1	< 0.001
	Dmax (Gy)	40.8 ± 3.6	42.5 ± 2.4	0.043

Abbreviations: SD, standard deviation; Gy, gray; PTV, planning target volume; Dmax, maximum dose; CI, Conformity Index; HI, Homogeneity Index; VX, the percentage of the organ volume that received X Gy or more; DX, dose received by the X% of the volume

### Work efficiency

**Fig. 3:**
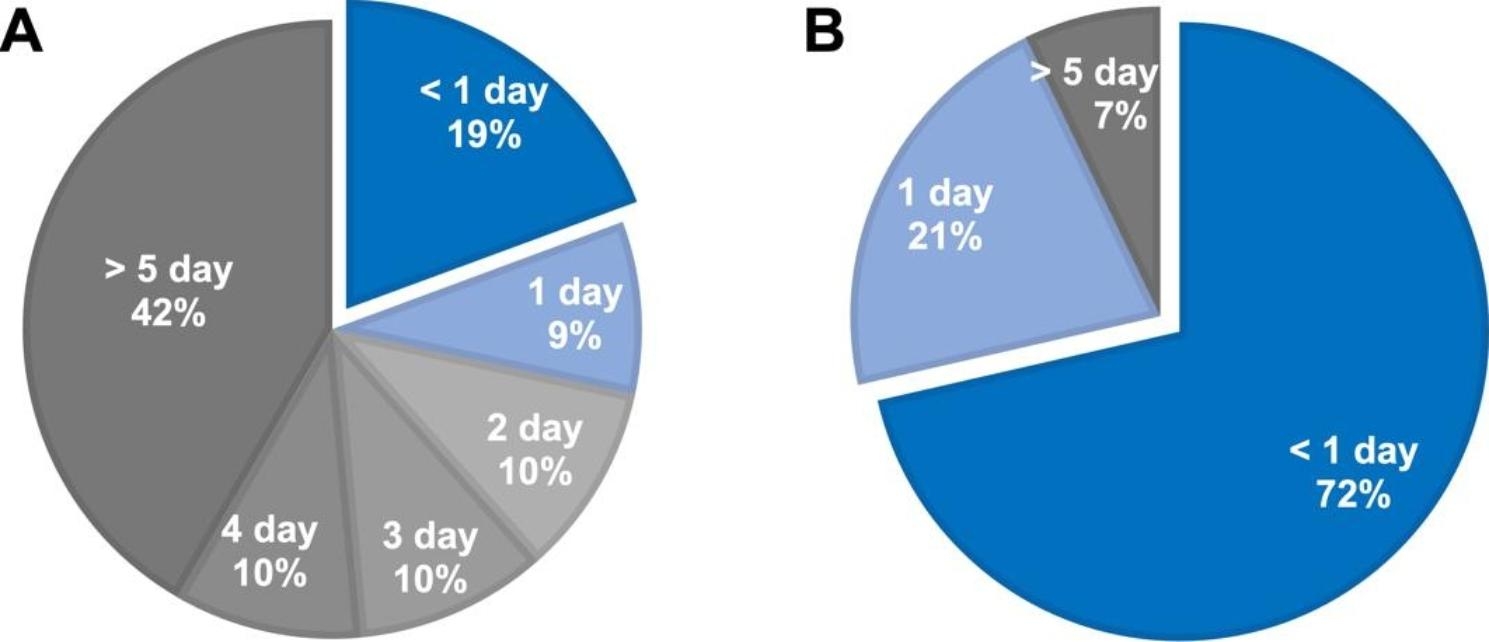
Improvement of work efficiency. **A**: The number of days required from the end of the physician’s contouring to the approval of the manual plan. **B**: The number of days required from the end of the physician’s contouring to the approval of the auto plan

Figure 3 shows the days required from the end of the physician’s contouring to the created VMAT plan approval. In the manual-VMAT, over 40% of cases required more than 5 days to approve the plan, and in only 19% of cases, approval was obtained on the same day that the physician’s contouring was completed. In the auto-VMAT, 93% of cases were approved within one day, and 72% of cases were approved on the same day that the physician’s contouring was completed.

### Treatment delivery parameters

Table 4 shows the gamma pass ratio, total MU and MCS in the auto-VMAT and manual-VMAT. All plans passed the patient-specific QA. And in both the initial and boost plans, there was no significant difference in the gamma pass ratio between the auto-VMAT and manual-VMAT (p = 0.675 and = 0.610, respectively). In both the initial and boost plans, total MU in the auto-VMAT was significantly higher than in the manual-VMAT (p < 0.001 and p = 0.012, respectively). In initial plans, MCS in the auto-VMAT were significantly lower than in the manual-VMAT (p = 0.031). In boost plans, MCS in the manual-VMAT were significantly lower than in the auto-VMAT (p = 0.012).

**Table 4:** Treatment delivery parameters

		Manual-VMAT (Mean ± SD)	Auto-VMAT (Mean ± SD)	*P* values
Initial plan				
	γpassing rate (%)	100.0 ± 0.15	100.0 ± 0.04	0.675
	Total MU	344.1 ± 43.9	406.3 ± 26.8	< 0.001
	MCS	0.31 ± 0.09	0.27 ± 0.04	0.031
Boost plan				
	γpassing rate (%)	100.0 ± 0.05	100.0 ± 0.04	0.610
	Total MU	279.8 ± 19.7	293.1 ± 17.3	0.012
	MCS	0.34 ± 0.10	0.41 ± 0.08	0.012

Abbreviations: MU, monitor unit; MCS, modulation complexity score; SD, standard deviation; VMAT, volumetric modulated arc therapy; criteria ofγpassing rate is dose difference of 3% and a distance to agreement of 2 mm (3%/2mm). The gamma pass rate of greater than 98% was the threshold to indicate robust delivery in this study

## Discussion

In this study, we developed a fully automatic feasible VMAT plan creation program that works within a commercial treatment planning machine for LARC. The developed program automatically sets the prescription dose, determines the irradiation geometry, inputs the dose constraint for optimization, creates the dose distribution, and dramatically reduced the time to approval. Because the additional ROIs for dose distribution adjustment in the development program change the shape in accordance with the shape of the PTV, it is possible to create a dose distribution with constant quality for each patient.

The auto-VMAT maintained high uniformity, reduced D2, and kept PTV coverage comparable to the manual-VMAT. All auto-VMATs passed the patient-specific QA. Of particular interest were the reductions in the mean dose, V30, and V40 in the bladder and small bowel with the auto-VMAT. This may be the effect of the additional ROIs and dose fall-off technique used in the auto-VMAT. In contrast, planners may have prioritized achieving PTV coverage constraints or may not have performed sufficient trial-and-error efforts to reduce OAR doses. Differences in planner experience and skills may have affected the results. Song et al. developed an automatic VMAT generation program for LARC using Pinnacle3 with the model for the Elekta Synergy accelerator and compared it with the manual-VMAT [18]. The authors reported that the auto-VMAT achieved quality equal to or better than the manual-VMAT and was particularly effective in reducing the small bowel dose. In our study, small bowel and bladder V30, V40, and mean dose were significantly lower in the auto-VMAT and similar to the previously reported results. CI in the auto-VMAT was lower than the manual-VMAT in our initial plan. This was probably because the restrictions for achieving PTV coverage set in the auto-VMAT were too strong. This may also be because of the steeper concave shape of the PTV compared with those reported in previous studies.

In comparing the time from completion of physician contouring to plan approval, the manual-VMAT required more than 2 days for plan approval in 72% of cases. In the auto-VMAT, 93% of cases obtained plan approval within 1 day. The auto-VMAT may have provided a drastic reduction of hands-on time for planning and the reduction of the review burden on physicians because of the standardization of plan quality. This made it possible to approve plans in a short period of time. In the future, vendor improvements to dose calculation algorithms and systems may yield further speedups. In addition, combining auto-contouring technology may reduce the overall planning times [27, 28].

The setting of the isocenter position, prescription dose, and the insertion of the virtual couch are manually performed by planners in the manual-VMAT. However, in radiotherapy, manual input and recognition errors can cause accidents. According to the TG100, it is difficult to detect errors in treatment planning, and these errors have a significant impact on patients [29]. In our development system, these setting inputs are performed fully automatically in the plan creation process, eliminating manual operation by humans and realizing a high level of medical safety. Unlike dose-volume histogram prediction–type automatic planning systems, our program is robust against differences in definitions of PTV shapes among facilities because the VMAT create section is dependent on the planner’s process. Furthermore, if the facility has a Raystation installed, our program can be implemented with a single text file.

This study has several limitations. First, the number of cases examined was small. Second, the manual-VMAT depended on the experience and skill of each planner. Third, in the manual-VMAT, when planners have multiple plan tasks, they may not start on low-priority plans immediately. This may be a bias that extends the time required for approval in the manual-VMAT.

## Conclusions

We developed a fully automatic feasible VMAT plan creation program for LARC. The auto-VMAT maintained target coverage while providing organs at risk dose reduction. The developed program dramatically reduced the time to approval.

## Data Availability

The datasets used and analyzed during the current study are available from the corresponding author on reasonable request.

## List of Abbreviations

auto-VMAT: Automated VMAT plan
CI: Conformity index
CTV: Clinical target volume
DVH: Dose-volume histograms
HI: homogeneity index
LARC: Locally advanced rectal cancer
manual-VMAT: Manual VMAT plan
MCS: Modulation complexity score
MU: Monitor unit
OAR: organs at risk
PTV: Planning target volume
QA: Quality assurance
VMAT: volumetric modulated arc therapy
